# Hyponatremia in infants with community-acquired infections on hospital admission

**DOI:** 10.1371/journal.pone.0219299

**Published:** 2019-07-05

**Authors:** Marta B. Mazzoni, Gregorio P. Milani, Silvia Bernardi, Ludovica Odone, Alessia Rocchi, Emanuela A. D’Angelo, Marco Alberzoni, Carlo Agostoni, Mario G. Bianchetti, Emilio F. Fossali

**Affiliations:** 1 Pediatric Emergency Department, IRCCS Foundation Cà Granda, Ospedale Maggiore Policlinico, Clinica De Marchi, University of Milan, Milan, Italy; 2 Pediatric Unit, Fondazione IRCCS Ca’ Granda Ospedale Maggiore Policlinico, Milan, Italy; 3 Department of Clinical Sciences and Community Health, Università degli Studi di Milano, Milan, Italy; 4 Pediatric Department of Southern Switzerland, Ospedale San Giovanni, Bellinzona, Switzerland; 5 Università della Svizzera Italiana, Lugano, Switzerland; Universita degli Studi di Parma, ITALY

## Abstract

Acute moderate to severe gastroenteritis is traditionally associated with hypernatremia but recent observations suggest that hypernatremia is currently less common than hyponatremia. The latter has sometimes been documented also in children with acute community-acquired diseases, such as bronchiolitis and pyelonephritis. We investigated the prevalence of dysnatremia in children with acute moderate severe gastroenteritis, bronchiolitis and pyelonephritis. This prospective observational study included 400 consecutive previously healthy infants ≥4 weeks to ≤24 months of age (232 males and 168 females): 160 with gastroenteritis and relevant dehydration, 160 with moderate-severe bronchiolitis and 80 with pyelonephritis admitted to our emergency department between 2009 and 2017. Circulating sodium was determined by means of direct potentiometry. For analysis, the Kruskal-Wallis test and the Fisher’s exact test were used. Hyponatremia was found in 214 of the 400 patients. It was common in gastroenteritis (43%) and significantly more frequent in bronchiolitis (57%) and pyelonephritis (68%). Patients with hyponatremia were significantly younger than those without hyponatremia (3.9 [1.6–13] versus 7.5 [3.4–14] months). The gender ratio was similar in children with and without hyponatremia. Hyponatremia was associated with further metabolic abnormalities (hypokalemia, hyperkalemia, metabolic acidosis or metabolic alkalosis) in gastroenteritis (71%) and pyelonephritis (54%), and always isolated in bronchiolitis. In conclusion, hyponatremia is common at presentation among previously healthy infants with gastroenteritis, bronchiolitis or pyelonephritis. These data have relevant consequences for the nutrition and rehydration management in these conditions.

## Introduction

Acute gastroenteritis with relevant dehydration is traditionally associated with hypernatremia [1–3]. However, some observations suggest that hypernatremia is currently less common than hyponatremia [1–3]. The latter electrolyte abnormality has sometimes been documented also in children with further community-acquired diseases, such as lower respiratory tract infections [4, 5].

Two preliminary reports from our institution documented that hyponatremia is very frequent on admission among otherwise healthy infants affected by moderate-severe bronchiolitis or pyelonephritis [6, 7]. The aim of the present study is to compare sodium, potassium and acid-base balance in the aforementioned infants with bronchiolitis or pyelonephritis and in infants with gastroenteritis.

### Patients and methods

The Ethical committee of Ca’ Granda Ospedale Maggiore Policlinico Milan (Italy) approved the study. Written consent obtained.

Since 2009, at the Pediatric Emergency Department of Ca’ Granda Ospedale Maggiore Policlinico, urea, creatinine, albumin, ionized sodium, chloride, potassium, glucose, pH and carbon dioxide pressure have been assessed on admission in all infants ≥4 weeks to ≤24 months of age with acute gastroenteritis and relevant dehydration, pyelonephritis and, more recently, in moderate-severe bronchiolitis [6, 7].

In infants with acute gastroenteritis, i.e. a stool frequency exceeding the child's usual number of daily bowel movements, the clinical dehydration score is calculated [8] based on general appearance (normal = 0; thirsty, restless or lethargic but irritable when touched = 1; drowsy, limp, cold or sweaty, comatose or not = 2), eyes (normal = 0; slightly sunken = 1; very sunken = 2), mucous membranes (moist = 0; sticky = 1; dry = 2), and tears (tears = 0; decreased = 1; absent = 2). Dehydration is considered relevant in cases with a score ≥5 [8].

The diagnosis of moderate-severe bronchiolitis and pyelonephritis is made according to our standard procedure. Briefly, bronchiolitis is diagnosed in infants with acute onset of worsening respiratory distress, cough and diffuse crackles on auscultation [6]. Respiratory rate (< 45/min = 0; 45–60/ min = 1; > 60/min = 2), O_2_-saturation in ambient air (> 95% = 0; 95–90% = 1; < 90% = 2), presence of thoracic retractions (none = 0; present = 1; present and associated with nasal flare = 2) and ability to feed (normal = 0; reduced = 1; strongly reduced = 2) are used. Bronchiolitis is considered moderate-severe in cases with a score ≥4 [6]. The diagnosis of pyelonephritis is made in cases with pathological pyuria, a positive urine culture, and clinical (body temperature) or laboratory (C-reactive protein, total white blood cell count) features of systemic inflammation [7].

Blood is collected anaerobically with minimal stasis [7]. Urea, creatinine and albumin are determined in plasma using a Cobas 8000 c702 analyzer. Ionized sodium, chloride, potassium, pH and carbon dioxide pressure (direct potentiometry) and glucose (amperometry) are assessed in whole blood in a GEM Premier TM 4000 analyzer [7]. A second blood sample is collected if potassium is ≥5.5 mmol/L and the lowest value is employed for clinical purposes. Bicarbonate is calculated from pH and carbon dioxide pressure [7].

Following normal value definitions are used: sodium 135–145 mmol/L; potassium 3.5–5.4 mmol/L; chloride 96–111 mmol/L; glucose ≤11.0 mmol/L; pH 7.36–7.44; carbon dioxide pressure 35–45 mm Hg, bicarbonate 20–27 mmol/L [7]. The diagnosis of metabolic acidosis is made in cases with hypobicarbonatemia and pH ≤7.40 (with or without hypocapnia), that of metabolic alkalosis in cases with hyperbicarbonatemia and pH ≥7.40 (with or without hypercapnia), that of respiratory acidosis in cases with hypercapnia and pH ≤7.40 (with or without hyperbicarbonatemia) and that of respiratory alkalosis in cases with hypocapnia and pH ≥7.40 (with or without hypobicarbonatemia) [7].

Considering that gastroenteritis and bronchiolitis are ≥2 times more frequent than pyelonephritis at our institution, we enrolled 400 consecutive infants, subdivided as follows: 160 with gastroenteritis and relevant dehydration, 160 with moderate-severe bronchiolitis and 80 with pyelonephritis. For the purposes of this study, we exclusively included previously apparently healthy infants.

Patients born with a gestational age <34 weeks or with recent medication potentially altering the electrolyte balance were not included [9]. Patients with signs of relevant circulatory compromise such as mottled or cool skin, very prolonged capillary refill, decreased intensity of distal pulses and severe arterial hypotension were also not included because circulatory compromise may per se alter acid-base and electrolyte balance [10]. The patients were also evaluated with respect to seizures and severely altered mental status, two signs of acute dysnatremia.

Results are given either as median and interquartile range or as frequency. For analysis, the Kruskal-Wallis test and the Fisher’s exact test were used. Statistical significance was assigned at P<0.05 [11].

## Results

**Fig 1:**
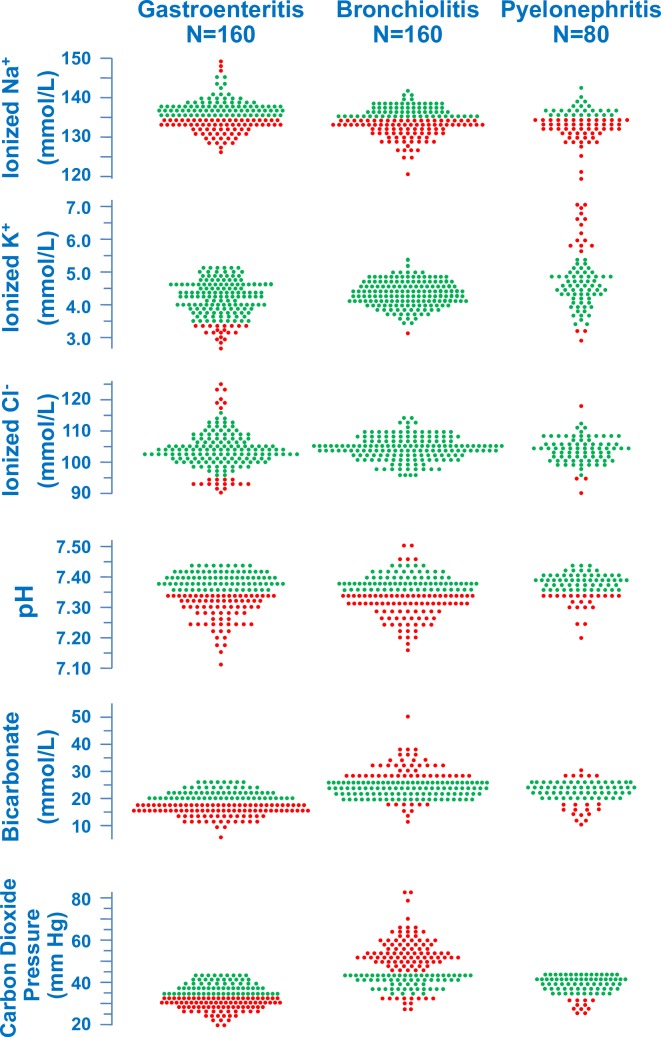
Electrolytes and acid-base balance on hospital admission. Ionized sodium, ionized potassium, ionized chloride, pH, bicarbonate and carbon dioxide pressure in infants ≥4 weeks to ≤24 months of age with gastroenteritis, bronchiolitis or pyelonephritis. Green circles denote normal and red symbols abnormally high or low values.

The patients were enrolled between 2009 and 2017. Clinical and laboratory data on hospital admission are given in Table 1 and Fig 1. The age was on the average higher in the group with gastroenteritis compared to bronchiolitis or pyelonephritis and in pyelonephritis compared to bronchiolitis. The study groups did not significantly differ with respect to gender. Urea and creatinine were higher in gastroenteritis compared to pyelonephritis or bronchiolitis.

**Table 1:** Clinical and laboratory data on hospital admission.

				Gastroenteritis	Bronchiolitis	Pyelonephritis	Gastroenteritis versus Bronchiolitis	Bronchiolitis versus Pyelonephritis	Gastroenteritis versus Pyelonephritis
N				160	160	80			
Male : Female (N)				87 : 73	93 : 67	52 : 28	P = 0.57	P = 0.33	P = 0.13
Age (months)				14 [9.5–17]	2.2 [1.5–4.3]	3.8 [1.9–6.6]	P<0.0001	P<0.05	P<0.0001
Plasma level									
	Urea (mmol/L)			4.0 [2.5–5.8]	2.5 [2.0–3.2]	2.7 [2.2–3.2]	P<0.0001	P = 0.43	P<0.0001
	Creatinine (μmol/L)			29 [25–34]	20 [15–26]	26 [19–31]	P<0.0001	P<0.0005	P<0.001
	Albumin (g/L)			45 [43–48]	43 [40–46]	42 [40–44]	P<0.0001	P<0.05	P<0.0001
Whole blood level									
	Ionized sodium								
		Value (mmol/L)		135 [133–137]	134 [132–136]	133 [132–135]	P<0.01	P = 0.29	P<0.0001
		Hyponatremia, N (%)		69 (43)	91 (57)	54 (68)	P<0.05	P = 0.13	P<0.005
		Hypernatremia, N (%)		3 (1.9)	0 (0.0)	0 (0.0)	P = 0.25	P = 1.0	P = 0.55
	Ionized potassium								
		Value (mmol/L)		4.1 [3.7–4.3]	4.4 [4.3–4.6]	4.8 [4.5–5.2]	P<0.0001	P<0.0001	P<0.0001
		Hypokalemia, N (%)		20 (13)	1 (0.6)	3 (3.8)	P<0.005	P = 0.11	P<0.05
		Hyperkalemia, N (%)		0 (0.0)	0 (0.0)	14 (18)	P = 1	P<0.0005	P<0.0005
	Ionized chloride								
		Value (mmmol/L)		102 [99–104]	104 [102–105]	103 [100–104]	P<0.01	P = 0.053	P = 0.29
		Hypochloremia, N (%)		15 (9.4)	0 (0.0)	3 (3.8)	P<0.0001	P<0.05	P = 0.19
		Hyperchloremia, N (%)		7 (4.4)	0 (0.0)	1 (1.3)	P<0.05	P<0.05	P = 0.27
	Glucose								
		Value (mmol/L)		4.4 [3.7–5.1]	5.5 [4.9–6.3]	5.3 [4.6–6.4]	P<0.0001	P = 0.63	P<0.0001
		Stress hyperglycemia, N (%)		0 (0.0)	0 (0.0)	0 (0.0)	P = 1.0	P = 1.0	P = 1.0
	Acid-base equilibrium								
		pH		7.36 [7.31–7.39]	7.34 [7.32–7.36]	7.38 [7.34–7.40]	P = 1.0	P<0.01	P = 0.055
		Carbon dioxide pressure (mm Hg)		34 [31–37]	47 [42–53]	40 [37–43]	P<0.0001	P<0.0001	P<0.0001
		Bicarbonate (mmol/L)		18 [16–21]	25 [23–27]	23 [20–25]	P<0.0001	P<0.0005	P<0.0001
		Metabolic acidosis, N (%)		94 (59)	0 (0.0)	14 (18)	P<0.0001	P<0.0001	P<0.0001
		Respiratory acidosis, N (%)		0 (0.0)	87 (54)	0 (0.0)	P<0.0005	P<0.0005	P = 1.0
		Metabolic alkalosis, N (%)		0 (0.0)	0 (0.0)	6 (7.5)	P = 1.0	P<0.005	P<0.005
		Respiratory alkalosis, N (%)		0 (0.0)	12 (7.5)	0 (0.0)	P<0.0005	P<0.01	P = 1.0

The table presents the characteristics of the 400 patients ≥4 weeks to ≤24 months of age (232 males and 168 females) affected by acute gastroenteritis with relevant dehydration, moderate-severe bronchiolitis or pyelonephritis. Results are given as median and interquartile range or as frequency and percentage.

Hypernatremia was detected exclusively in approximately 2% of the infants with gastroenteritis. Hyponatremia was detected in more than half (214, 54%) of the 400 patients and was significantly more common in bronchiolitis and pyelonephritis compared to gastroenteritis. Signs of acute onset dysnatremia were never observed. Patients with hyponatremia were significantly younger than those without hyponatremia (3.9 [1.6–13] versus 7.5 [3.4–14] months, P<0.001). The gender ratio was similar in children with (126 males and 88 females) and without (106 males and 80 females) hyponatremia.

A tendency to hypokalemia was common in gastroenteritis, whilst a tendency to hyperkalemia was common in pyelonephritis. A tendency to dyschloremia was rather common in gastroenteritis, uncommon in pyelonephritis and absent in bronchiolitis. Glucose level was lower in gastroenteritis compared with bronchiolitis and pyelonephritis. Stress hyperglycemia was never observed. Metabolic acidosis was common both in pyelonephritis and especially in gastroenteritis: it was associated with hyperkalemia (5 out of 14 cases; 36%) in the former and with hypokalemia (13 out of 94 cases; 14%) in the latter disease. Respiratory acidosis was common exclusively in bronchiolitis. Metabolic and respiratory alkalosis were rather uncommon. Hyponatremia was associated with further metabolic abnormalities such as hypokalemia, hyperkalemia, metabolic acidosis or metabolic alkalosis in approximately one third of the cases (Table 2): it was mostly combined in gastroenteritis and pyelonephritis and always isolated in bronchiolitis.

**Table 2:** Isolated and combined hyponatremia.

	Hyponatremia	
	Isolated	Combined
Gastroenteritis, N (%)	20 (29)	49 (71)
Bronchiolitis, N (%)	91 (100)	0 (0.0)
Pyelonephritis, N (%)	25 (46)	29 (54)
**Total, N (%)**	**136 (64)**	**78 (36)**

Metabolic abnormalities (hypokalemia, hyperkalemia, metabolic acidosis or metabolic alkalosis) associated with hyponatremia in 214 patients ≥4 weeks to ≤24 months of age (126 male and 88 female patients) affected by acute gastroenteritis with relevant dehydration, moderate-severe bronchiolitis or pyelonephritis.

## Discussion

Hyponatremia is increasingly observed (currently approximately 15%) in subjects ≥55 years of age presenting to an emergency department [9, 12]. Risk factors for this dyselectrolytemia include female gender, chronic heart or kidney disease, medication with thiazides, blockers of the renin-angiotensin system or serotonin reuptake inhibitors and genetic factors [9, 12, 13]. The present study confirms that asymptomatic hyponatremia is very common (slightly less than 50%) at presentation among previously healthy infants ≥4 weeks to ≤24 months of age with gastroenteritis and relevant dehydration [1–3] and points out that its frequency is even higher in infants affected by moderate-severe bronchiolitis (slightly less than 60%) or pyelonephritis (slightly less than 70%). In this setting, hyponatremia affects both genders with a preference for children ≤12 months of age.

The mechanisms underlying hyponatremia have to be briefly discussed. First. Creatinine and urea levels were higher in gastroenteritis than in bronchiolitis and pyelonephritis, suggesting a more relevant fluid depletion in the former condition [14]. Second. Hyponatremia was always isolated in bronchiolitis suggesting that inappropriate antidiuresis, which habitually presents without associated metabolic acid-base or potassium abnormalities, might underlay hyponatremia exclusively in this condition [15]. Third. Until the 1980s, hyper- and normo-natremia were common and hyponatremia rare in gastroenteritis [1]. In the following decades, hypernatremia almost disappeared whilst the prevalence of hyponatremia increased [1]. Two factors likely explain this shift: the resurgence of breastfeeding and formulas having less salt, and the present attitude towards gastroenteritis, with early and fast reintroduction of mostly hypotonic fluids [1, 16]. Similar factors might explain, at least in part, the development of this dyselectrolytemia in bronchiolitis and pyelonephritis as well [6, 7]. Similarly, currently recommended low salt ingestion for the general population might perhaps also contribute [9, 12]. Finally, considering that genetic factors increase the risk of thiazide-induced hyponatremia [13], we speculate that community-acquired infections might be complicated by hyponatremia especially in predisposed subjects.

Metabolic acidosis is common both in acute gastroenteritis and pyelonephritis and often associated with hypokalemia in the former and especially hyperkalemia in the latter. In gastroenteritis, acidosis and hypokalemia are caused by intestinal loss of organic acid anions and potassium [1, 3]. In infants with pyelonephritis, acidosis and hyperkalemia result from transient renal resistance to aldosterone [7, 17]. In both conditions, intensive inflammatory response with high levels of mediators such as interleukin-1 and tumor necrosis factor might play a crucial pathogenic role [18, 19].

Our findings have relevant consequences for the nutrition and rehydration management. First. It is recommended, for children affected by an acute infection, to pursue breastfeeding or other milk feeds and increase fluid intake. Because sodium level is very low in soft drinks, such as “flat” cola, fluids containing more sodium might be preferred [1, 3]. Second. Our data further highlight the advantages of parenteral isotonic fluids and the risks of hypotonic solutions [20, 21]. Recent observations suggest that isotonic fluids correct hyponatremia both in cases without and with laboratory features consistent with inappropriate antidiuresis [22].

The strengths of this study rely on inclusion of a relative high number of consecutive patients and on the assessment of sodium by direct potentiometry, as recommended by laboratory medicine specialists [23, 24]. Our investigation has some limitations, though. First, it did not specifically address burden, clinical course and long-term outcomes of infants with hyponatremia. Second, it did not investigate the mechanisms underlying hyponatremia. Third, the role of the microorganisms causing gastroenteritis, bronchiolitis or pyelonephritis was not considered. Fourth, recommended preventive and therapeutic strategies arise from authors’ opinions.

The term “hyponatremia epidemic” has been used to denote the high prevalence of hyponatremia in adults presenting to an emergency department [9, 12]. This term might also be applied for the prevalence of this dyslectrolytemia in previously healthy infants affected by acute community acquired infections.
